# Advanced HIV Disease at First Diagnosis in South Brazil Extended Beyond Traditionally Targeted Populations: A 10‐Year Hospital‐Based Study (2015–2024)

**DOI:** 10.1155/arat/6671976

**Published:** 2026-07-15

**Authors:** Christopher Justin Hernandez, Pedro Moreno Fonseca, Andressa Noal, Maxwell Zywica, Mary Catherine Cambou, Ivana Rosângela dos Santos Varella, Breno Riegel Santos, Marineide Gonçalves de Melo, Karin Nielsen-Saines

**Affiliations:** ^1^ University of California Los Angeles David Geffen School of Medicine, Los Angeles, California, USA, ucla.edu; ^2^ Hospital Conceicao, Porto Alegre, State of Rio Grande do Sul, Brazil

**Keywords:** advanced HIV disease, gender and sexual minorities, heterosexual, HIV risk factors, late HIV diagnosis, south Brazil

## Abstract

**Background:**

Advanced HIV disease (AHD) remains a major contributor to HIV‐related morbidity and mortality. While HIV screening strategies in Brazil emphasize populations traditionally considered at high risk, a significant number of individuals without previous HIV infection present with AHD despite lacking recognized risk profiles. This study aimed to define the sociodemographic and behavioral characteristics of patients presenting with advanced HIV at diagnosis, with the goal of identifying gaps in current HIV screening efforts.

**Methods:**

We conducted a retrospective cohort study of patients hospitalized in the infectious diseases ward of a tertiary referral hospital between January 2015 and December 2024. We included individuals previously unaware of their HIV status who met World Health Organization (WHO) criteria for AHD, defined as CD4 count < 200 cells/mm^3^ and/or a WHO Clinical Stage 3 or 4 condition. Sociodemographic characteristics, behavioral risk factors, and clinical data were abstracted from medical records and standardized epidemiologic intake forms. Descriptive analyses compared characteristics across sexuality and gender groups.

**Results:**

Among 1615 unique patients hospitalized with AHD, 407 (25%) were newly diagnosed with HIV during hospitalization. The median CD4 count at diagnosis was 53 cells/mm^3^ (IQR 27–110), reflecting profound immunosuppression. Most patients identified as heterosexual men (49%), while only 10% identified as sexual or gender minorities. Fewer than one‐quarter (24%) had psychosocial risk factors commonly emphasized in HIV screening strategies. Heterosexual men were predominantly employed, married or partnered, and had children, while women and sexual and gender minority patients were older and younger, respectively, and reported fewer risk factors. Injection drug use history and homelessness were rare across all groups.

**Conclusions:**

AHD at first diagnosis was not concentrated within traditionally recognized high‐risk populations. The diversity of patients presenting with AHD at first diagnosis suggests that reliance solely on traditional risk‐based screening strategies may be insufficient to ensure timely detection. Expanded routine, opt‐out HIV testing across healthcare settings, as well as indicator‐based testing, self‐testing, and pharmacy‐based testing may help reduce missed opportunities for diagnosis and prevent severe immunosuppression.

## 1. Introduction

Advanced HIV disease (AHD) is defined by the World Health Organization (WHO) as HIV infection with a CD4 T lymphocyte count (CD4 count) < 200 cells/mm^3^ and/or the presence of a WHO Clinical Stage 3 or 4 condition, including severe symptoms or opportunistic infections [1]. Untreated HIV typically progresses to AHD over several years (often within 5–10 years), suggesting that individuals diagnosed at this stage likely experienced multiple missed opportunities for screening and ART initiation [2]. AHD is associated with a higher prevalence of neoplasms, metabolic and endocrine derangements, and neurocognitive disorders, such as HIV‐associated dementia [3]. Further, individuals diagnosed at an advanced stage have a life expectancy of approximately 6.6 years shorter than those diagnosed at earlier stages [4]. A study in the United Kingdom found that life expectancy at age 20 depended on the CD4 count when ART was initiated: 37.9 years for those with CD4 counts less than 100, 41 years for those with CD4 counts between 100 and 199, and 53.4 years for those with CD4 counts between 200 and 350 [5]. The excessive mortality observed among individuals with AHD reflects delayed diagnosis and treatment initiation, occurring after substantial immune dysfunction and disease burden [6]. Furthermore, AHD is often accompanied by elevated viral loads (VLs) that substantially increase the risk of transmission, particularly among those unaware of their status and therefore not on antiretroviral therapy (ART) or virally suppressed [7]. For these reasons, finding people living with HIV (PLH) before the onset of AHD is essential in preventing morbidity and mortality as well as averting new cases.

Despite significant global efforts to promote early HIV detection and ART initiation, many individuals continue to present with AHD, particularly in regions with socioeconomic disparities and structural barriers to healthcare access [8]. Surveillance data indicate that Brazil has continued to report tens of thousands of new HIV infection diagnoses each year in recent years, with 46,495 cases in 2023, a 4.5% increase compared to 2022 [9]. Of these new cases, 63% were among individuals who self‐identified as Black or Mixed and 54% were among men who have sex with men (MSM). Young adults aged 20–29 years represented the largest proportion of new diagnoses. Significant regional disparities in HIV incidence and outcomes continue to shape the epidemic in Brazil. Between 2005 and 2020, the Southeast and South regions accounted for 42.7% and 20.9% of AIDS cases, respectively [10]. Further, Porto Alegre, the capital of Rio Grande do Sul, has consistently ranked among the Brazilian capitals with the highest AIDS‐related mortality rates, with rates multiple times higher than the national average, exceeding 20 per 100,000 inhabitants in recent years [9]. Interestingly, south Brazil has demonstrated transmission patterns that are unique compared to the rest of the country. A recent state‐wide serological household survey conducted in the state of Rio Grande do Sul demonstrated that HIV prevalence was strongly associated with lower educational attainment, lower household income, and residence in socially vulnerable neighborhoods, and identified substantial heterosexual transmission, consistent with prior studies suggesting a more generalized epidemic in the region [11]. While these data describe HIV incidence and prevalence, less is known about the sociodemographic profile of individuals who present with AHD at the time of first diagnosis in high‐burden regions of Brazil. Characterizing this population may inform strategies to reduce undiagnosed infection and missed opportunities for earlier detection.

The present study examines the sociodemographic and behavioral characteristics of patients diagnosed with HIV who met criteria for AHD at hospitalization in a tertiary referral center in Porto Alegre, Brazil. By describing the profile of individuals presenting with late diagnosis, we aim to identify populations underserved by current testing strategies and inform targeted public health interventions in this high‐burden setting.

## 2. Methods

### 2.1. Study Design and Setting

We conducted a retrospective cohort study using hospital records from a tertiary‐level public hospital and HIV referral institution in Porto Alegre, Rio Grande do Sul (RS), South Brazil. Medical records were evaluated for all patients hospitalized and managed by the infectious diseases ward between January 1, 2015, and December 31, 2024. The infectious diseases ward receives patients referred from the emergency department, outpatient clinics, and other inpatient services when an infectious condition with complex medical needs becomes the primary concern. Although a substantial proportion of patients admitted to this ward are PLH, the service also manages a broad spectrum of infectious conditions unrelated to HIV. The hospital utilizes an integrated electronic medical record (EMR) system in which clinical, laboratory, and social history data are documented by attending physicians, resident physicians, social workers, and nursing staff. Epidemiologic intake forms are routinely completed at the time of hospitalization as part of institutional surveillance protocols required for compulsory notification of reportable conditions [9].

### 2.2. Study Population

For the present analysis, we focused specifically on individuals who met criteria for AHD at the time of first HIV diagnosis. The WHO criteria for AHD are defined as an HIV infection with either a CD4 count < 200 cells/mm^3^ or the presence of a WHO Clinical Stage 3 or 4 condition, regardless of CD4 count [1]. WHO Clinical Stage 3 or 4 conditions include severe HIV‐related symptoms and/or opportunistic infections and malignancies indicative of advanced immunosuppression. First HIV diagnosis was defined as the earliest documented positive HIV test during the study period, with no evidence of prior ART dispensation recorded in national laboratory databases.

### 2.3. HIV Diagnosis

HIV infection was diagnosed according to the Brazilian Ministry of Health testing algorithm in effect during the study period [12]. Initial screening was performed using rapid immunochromatographic assays approved for use in the public health system. Reactive rapid test results were confirmed using enzyme‐linked immunosorbent assays (ELISAs) or immunoblot assays detecting HIV‐1 antigens (p24, gp41, gp120, gp160) and HIV‐2 antigen gp36. HIV‐specific clinical parameters were extracted from the EMR, including date of diagnosis, VL, and CD4+ T‐cell count at presentation. To confirm laboratory values and ART history, exact record linkage was performed using each patient’s unique Sistema Único de Saúde (SUS) identification number to cross‐reference records with the Sistema de Controle de Exames Laboratoriais (SISCEL), the national database that captures CD4 counts, HIV VLs, and ART dispensation data in Brazil [13]. Identifiers were accessed solely for linkage purposes and were removed prior to statistical analysis.

### 2.4. Sociodemographic Data

Sociodemographic and risk factor data were abstracted from two sources: (1) medical and social worker notes that included this information at length and (2) standardized epidemiological intake forms routinely completed at the time of hospitalization following surveillance protocols. Patients for whom risk factor information was unavailable due to inability to communicate (e.g., severe illness or coma) and an absence of collateral sources were coded as unknown. For this study, age was treated as a continuous variable. Race or ethnicity was categorized according to hospital records as White, Black, or Mixed. There were no other races or ethnicities that patients identified with in this study. Marital status was defined as married/partnered or not (including single, divorced, or widowed). Information on whether patients had children was also retrieved.

Sexual orientation and gender identity were assessed by self‐report, reflecting the study’s focus on the social implications of openly identifying as a sexual or gender minority (SGM). Heterosexual males (HM) and heterosexual females (HF) were those who identified as men who have sex with women and women who have sex with men, respectively. When sexual orientation or gender identity was not recorded on epidemiologic intake forms, clinicians’ notes were reviewed for descriptors such as MSM, gay, bisexual, or transgender which were used to classify patients belonging to an SGM group. Patients for whom no information on sexuality was available were categorized as “Not Disclosed/Unknown” (ND). Because ND reflects missing documentation rather than a patient’s identity, this group is presented separately.

Homelessness prior to initial hospitalization was identified by one of the following means: (1) if clinical notes by the street clinic/“Consultório na Rua” were present in the medical records, (2) if the patient self‐reported being unhoused at any point in the medical records, or (3) if the local epidemiological intake form indicated homelessness. Substance use, including tobacco, alcohol, injection drug, cocaine, or crack use were assessed from the intake form or from psychiatry and infectious disease notes. These data were dichotomized as never user or lifetime user as the degree of abstinence was not clear from medical records.

We created a composite “risk proxy” variable representing the presence of ≥ 1 structural or substance‐related characteristic commonly included in risk‐based HIV screening strategies commonly emphasized in Brazil (homelessness, incarceration, sex work, injection drug use, crack use, or cocaine use). Because this composite variable aggregates various characteristics of differing risk profiles, it should be interpreted as a marker of the presence of any traditionally recognized risk factor rather than a unified risk group.

### 2.5. Statistical Analysis

Sociodemographic characteristics were compared across sexual orientation and gender identity groups. Chi‐square tests of independence (if all *n* > 5) or Fisher’s exact tests (if at least one *n* ≤ 5) were used to determine associations between reported sexuality and socio‐demographic and clinical information. Reported *p* values are derived from unadjusted bivariate tests and are intended to describe differences in observed distributions across groups rather than to infer independent associations. Given the descriptive nature of the analysis and the absence of a comparator group diagnosed earlier in infection, these findings should be interpreted as characterizing the population presenting with AHD at first diagnosis rather than identifying predictors of late presentation. A two‐sided alpha level of 0.05 was used to define statistical significance. Individuals with missing data were excluded from the corresponding analyses. All analyses were conducted using STATA version 14 (College Station, TX, USA). Figures were generated using the RStudio platform (R Foundation for Statistical Computing).

### 2.6. Ethical Considerations

The study was reviewed and approved by the Research Ethics Committee of the Conceição Hospital Group (Comitê de Ética em Pesquisa do Grupo Hospitalar Conceição, CEP‐GHC) under protocol number 23‐141, issued on September 12, 2023. As this was a retrospective analysis with no direct contact with participants and no identifying information, the requirement for informed consent was waived by the ethics committee.

## 3. Results

**FIGURE 1:**
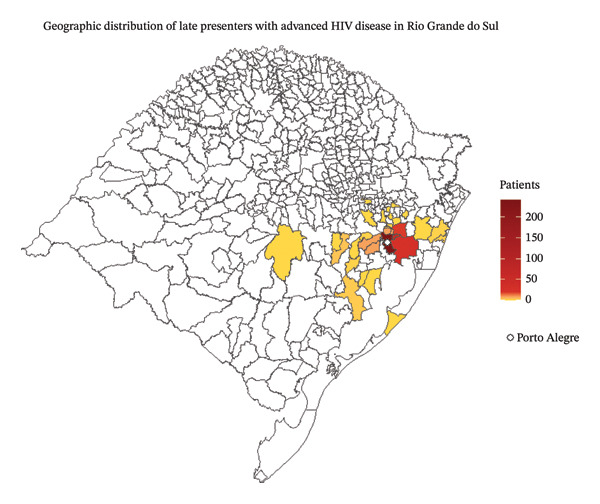
Geographic distribution, by municipality, of patients presenting with AHD at first diagnosis in Rio Grande do Sul, Brazil.

**FIGURE 2:**
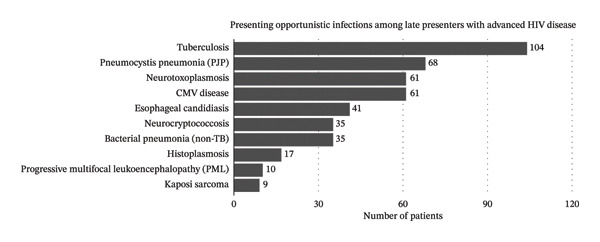
Presenting opportunistic infections among patients hospitalized with advanced HIV disease.

During the study period, there were 3636 HIV‐related hospitalizations comprising 2389 unique PLH. Of these, 2366 hospitalizations were due to AHD, encompassing 1615 unique PLH. In total, 407 (25%) patients with AHD were newly diagnosed with HIV during hospitalization (Table 1). There were 198 HM (49%), 142 HF (35%), 42 SGM (10%), and 25 ND (6%). SGM patients included 33 MSM, 5 transgender women, 3 bisexual men, and 1 bisexual woman. Most patients came from the urban and metropolitan regions of Rio Grande do Sul; however, many surrounding municipalities were represented by this sample (Figure 1). The overall CD4 median count at time of admission was 53 (IQR 27–110). Between 2015 and 2024, the median CD4 counts were below 100 cells/mm^3^, with substantial variability and overlapping interquartile ranges across calendar years (Supporting Figure1). No sustained temporal improvements in CD4 counts at presentation were observed. The most common causes of hospitalization were tuberculosis, pneumocystis pneumonia, neurotoxoplasmosis, and bacterial pneumonia (Figure 2).

**TABLE 1:** Demographic characteristics among patients hospitalized with a late diagnosis of HIV that met advanced HIV disease criteria at the time of presentation.

Characteristic	Total (*n* = 407)	Heterosexual male (*n* = 198)	Heterosexual female (*n* = 142)	Sexual/gender minority (*n* = 42)	Unknown (male) (*n* = 25)	*p* value^a^
Age						
Median (IQR)	41 (34, 50)	42 (36, 52)	44 (35, 54)	31 (26, 38)	38 (33, 45)	0.001
Race						
White	274 (67.3)	141 (71.2)	92 (64.8)	27 (64.3)	14 (56.0)	0.477
Black	103 (25.3)	45 (22.7)	37 (26.1)	11 (26.2)	10 (40.0)	
Mixed	30 (7.4)	12 (6.1)	13 (9.2)	4 (9.5)	1 (4.0)	
Employment (*n* = 404)						
Unemp/Ret/Stud	114 (28.2)	30 (15.2)	66 (47.1)	11 (26.2)	7 (28.0)	0.001
Employed	290 (71.8)	167 (84.7)	74 (52.9)	31 (73.8)	18 (72.0)	
Schooling (*n* = 393)						
Lower than HS	283 (72.0)	147 (77.4)	95 (69.9)	25 (59.5)	16 (64.0)	0.047
HS or higher	110 (27.8)	43 (22.6)	41 (30.1)	17 (40.5)	9 (36.0)	
Region						
Urban	240 (58.9)	109 (55.1)	84 (59.2)	32 (76.2)	15 (60.0)	0.340
Metropolitan	137 (33.7)	72 (36.4)	48 (33.8)	9 (21.4)	8 (32.0)	
Rural/interior	30 (7.4)	17 (8.6)	10 (7.0)	1 (2.3)	2 (8.0)	
Marital status (*n* = 399)						
Single/divorced	208 (52.1)	79 (40.5)	84 (60.4)	22 (52.3)	23 (100.0)	0.001
Married/partnered	191 (47.9)	116 (59.5)	55 (39.6)	20 (47.6)	0 (0.0)	
Children (*n* = 374)						
No	129 (34.5)	51 (28.3)	26 (19.3)	33 (82.5)	19 (100.0)	0.001
Yes	245 (65.5)	129 (71.7)	109 (80.7)	7 (17.5)	0 (0.0)	
Age of children (*n* = 233)						
≥ 18 years	116 (49.8)	55 (45.8)	59 (55.7)	2 (28.6)	0 (0.0)	0.176
< 18 years	117 (50.2)	65 (54.2)	47 (44.3)	5 (71.4)	0 (0.0)	
Risk factors						
Homelessness (*n* = 396)	18 (4.5)	11 (5.6)	3 (2.1)	2 (5.0)	2 (8.3)	0.357
Sex work (*n* = 396)	3 (0.7)	1 (0.5)	1 (0.5)	1 (2.4)	0 (0.0)	0.597
History of imprisonment (*n* = 396)	10 (2.5)	8 (4.0)	1 (0.7)	0 (0.0)	1 (4.0)	0.158
Tobacco use (*n* = 392)						
Lifetime use	169 (43.1)	92 (47.9)	50 (36.2)	16 (41.0)	11 (47.8)	0.191
Alcohol use (*n* = 393)						
Lifetime use	101 (25.7)	68 (35.4)	15 (10.8)	6 (15.4)	12 (52.2)	0.001
Crack cocaine (*n* = 394)						
Lifetime use	55 (13.9)	33 (17.2)	11 (7.9)	4 (10.0)	7 (30.4)	0.009
Powder cocaine (*n* = 395)						
Lifetime use	57 (14.4)	42 (21.9)	7 (5.0)	2 (5.0)	6 (26.1)	0.001
Injection drug use (*n* = 395)						
Lifetime use	5 (1.3)	4 (2.0)	0 (0.0)	0 (0.0)	1 (4.0)	0.180
Risk proxy^b^ (*n* = 399)						
≥ 1 risk factor	97 (24.3)	66 (33.3)	17 (11.9)	5 (11.9)	9 (36.0)	0.001

^a^ *p* values are from unadjusted bivariate tests and describe differences across groups; they are not intended to infer independent associations or predictors.

^b^Composite of any crack or cocaine use, injection drug use, history of housing instability, sex work, or imprisonment.

**FIGURE 3:**
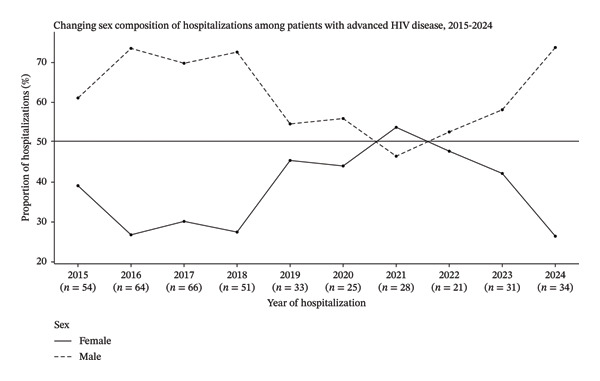
Changing sex composition of hospitalizations among patients diagnosed with advanced HIV disease during hospitalization, 2015–2024.

Absolute number of AHD‐at‐diagnosis admissions fluctuated, from 54 to 66 admissions annually between 2015 and 2017 to a nadir of 21 hospitalizations in 2022 before increasing to 31 in 2023 and 34 in 2024 (Figure 3). During the earlier years, hospitalizations were largely among male patients, who accounted for more than 60% of admissions. Beginning in 2019, the proportion of female patients increased, exceeding 50% in 2021. In subsequent years, the sex composition shifted again toward male predominance.

Table 1 lists the sociodemographic characteristics of the cohort, stratified by sexual orientation. The median age was 41 years (IQR 34–50), with significant age differences across groups (*p* = 0.001). SGM individuals were younger (31, IQR 26–38) compared with HM (42, IQR 36–52) and HF (44, IQR 35–54), while patients with ND identity had a median age of 38 years (IQR 33–45). Overall, 67% of patients identified as White, 25% as Black, and 7% as Mixed. Most patients (72%) reported being employed at the time of hospitalization. Employment was highest among HM (85%), while HF had substantially higher rates of unemployment, retirement, or student status (47%). Lower educational attainment was common, with 72% reporting schooling below the high school level, and this differed significantly across groups (*p* = 0.047), with SGM having the highest proportion of high school education or higher (41%). Most participants resided in urban (59%) or metropolitan (34%) areas. Regarding marital status, just under half (48%) were married or partnered, while 52% were single or divorced; marital status varied significantly by group (*p* = 0.001), with HM more frequently married or partnered (60%) than HF (40%) and SGM (48%). Most patients (66%) reported having children, although this differed markedly across groups (*p* = 0.001): most HM (72%) and HF (81%) had children, whereas most SGM (83%) reported having no children.

Homelessness (4.5%), sex work (1%), and history of incarceration (3%) were infrequently documented and did not differ significantly by sexuality group. Lifetime tobacco use was reported by 43% of patients. Alcohol use differed significantly (*p* = 0.001), being most prevalent among HM (35%) and least common among HF (11%). Lifetime crack cocaine use (14%) and powder cocaine use (14%) also varied significantly by group, with the highest prevalence observed among HM (*p* = 0.009). Notably, injection drug use was rare (1%) and did not differ significantly between groups. Overall, 24% of patients had at least one characteristic included in the composite risk proxy, with higher prevalence among HM (33%) and ND patients (36%) compared with HF (12%) and SGM patients (12%) (*p* = 0.001).

## 4. Discussion

In this retrospective cohort of patients hospitalized with AHD in Porto Alegre, nearly one‐quarter of individuals were previously unaware of their HIV status. The most common reasons for hospitalization among patients with AHD at first diagnosis include severe respiratory and neurological OIs associated with profound immunosuppression, with tuberculosis as the leading cause. Patients were predominantly from urban and metropolitan areas of Rio Grande do Sul, reflecting the hospital’s role as a tertiary referral center at the epicenter of the local epidemic, although surrounding municipalities also bore a substantial burden of disease. Notably, most patients identified as heterosexual men and women and had a relatively low prevalence of risk factors commonly emphasized in HIV screening strategies, such as stimulant use, homelessness, sex work, or prior imprisonment. It is important to note that these factors were ascertained from routine clinical and epidemiologic documentation and may be under‐reported if not disclosed by patients or if deprioritized during clinical encounters. Approximately one‐tenth of patients identified as SGM. Together, these findings highlight that patients presenting with AHD at first diagnosis extend beyond traditionally recognized key populations.

The median CD4+ count at diagnosis was approximately 50 cells/mm^3^, indicating that profound immunosuppression at the time HIV infection was first identified. Hospitalizations were predominantly driven by severe OIs, including pneumocystis pneumonia, tuberculosis, bacterial pneumonia, neurocryptococcosis, and neurotoxoplasmosis. These findings indicate that HIV diagnosis is commonly occurring after progression to illness requiring inpatient care, rather than during earlier asymptomatic phases of infection when optimal health outcomes can be preserved [5, 6, 8]. Because progression from HIV acquisition to advanced immunosuppression typically occurs over several years, the severity of disease at presentation is consistent with advanced immunosuppression at presentation, suggesting delayed diagnosis [2, 14]. Interviewer‐directed and qualitative studies among hospitalized patients presenting with newly diagnosed AHD that explicitly assess prior access to healthcare, HIV testing, and risk perception can help better understand where outreach strategies can be better informed.

The patient population hospitalized with AHD at first diagnosis was diverse and not limited to key populations. Although most individuals identified as White, Black patients were disproportionately represented relative to the general population of Rio Grande do Sul, consistent with well‐documented racial disparities in HIV prevalence in South Brazil [10, 15]. Heterosexual men accounted for nearly half of the patient population, were mostly married, employed, and with children. In this study, they had the highest prevalence of risk factors associated with HIV risk. Women also comprised a substantial proportion of hospitalizations and were on average older, more likely to be unemployed or retired, and more likely to have children. Most women in the cohort were older and reported having children, indicating that AHD at first diagnosis also occurs among women outside the typical context of antenatal HIV testing. Our findings are consistent with regional epidemiologic reports describing substantial heterosexual transmission in south Brazil [11, 16]. Older heterosexual adults who are married or partnered are often perceived as lower risk yet still account for a substantial proportion of people with AHD at first diagnosis. Some evidence demonstrates that marriage and stable partnerships do not necessarily protect from HIV acquisition and has been associated with lower perceived HIV risk and reduced engagement in preventive behaviors in prior studies [17]. Brazilian qualitative studies similarly suggest that trust in fixed partnerships may reduce perceived HIV vulnerability and contribute to delayed diagnosis [18–20].

About a tenth of patients were SGM in this cohort and were younger on average. Despite broad access to HIV testing and treatment in Brazil, MSM remain disproportionately affected by HIV, accounting for more than half of new HIV cases annually [9]. Within MSM communities, prevalence estimates are approximately 12% and emerging evidence suggests an increasing burden among younger MSM [21–23]. Furthermore, a recent study on the HIV care cascade in Brazil found that less than half of MSM who are living with HIV know about their status [24]. However, in our study, most presenting with AHD did not identify as MSM or SGM, which may reflect differences in testing patterns or healthcare engagement among SGM populations. SGM individuals were younger on average; however, the reasons for this difference cannot be determined from these data, although prior studies have suggested earlier sexual debut and cumulative exposure to risk as potential contributing factors [22, 23]. In any case, SGM still represent a significant portion of people who are not having an early diagnosis. Current outreach strategies would also be well informed by studies that examine barriers to testing among MSM in South Brazil.

The Brazilian Ministry of Health recommends targeted HIV testing among populations at heightened risk, including MSM, transgender individuals, people who use drugs, and sex workers, as well as testing individuals with clinical indications such as sexually transmitted infections or symptoms suggestive of HIV infection [12]. Although not explicitly listed in formal screening guidelines, factors such as housing instability and history of imprisonment are associated with elevated HIV risk [25–27]. However, the metropolitan region of Porto Alegre has a generalized epidemic affecting both men and women, including individuals who reported only one sexual partner in the prior year, no same‐sex intercourse, and no illicit drug use [11, 16]. Understanding which populations are presenting to the hospital with AHD without a prior diagnosis of HIV helps tailor outreach and improve early detection. The diversity of demographic and social characteristics observed in this cohort suggests that risk‐based approaches alone may not capture all individuals who ultimately present with advanced disease. Late presentation likely reflects a multifactorial process, including low perceived risk, missed testing opportunities in healthcare settings, structural barriers to care, stigma, and gaps in linkage or retention. Because our study did not include individuals diagnosed earlier in the course of infection, we are unable to determine which mechanisms predominate in this population. Our data support the consideration of expanded routine, opt‐out HIV testing across healthcare settings, an approach that has been associated with increased testing uptake in other settings [28–30]. In high‐burden regions such as Porto Alegre, it is critical to examine whether risk‐based screening strategies are sufficient to identify individuals before progression to advanced disease and whether a system‐level adoption of routine HIV testing represents a viable strategy to identify infection and reduce avoidable morbidity, mortality, and onward transmission.

### 4.1. Limitations

As a single‐center, hospital‐based study conducted at a tertiary referral institution, findings may be subject to selection bias. Patients admitted to this center may represent more severe or complex cases of AHD and may not fully reflect patterns among individuals diagnosed in outpatient or community settings. As such, generalizability may be limited. A second limitation regards behavioral and social variables which were derived from clinical documentation and standardized epidemiologic intake forms. These data may be subject to reporting bias, particularly for socially sensitive information such as substance use, housing instability, incarceration history, sex work history, and sexual orientation. Anticipated stigma and concerns about confidentiality may have led some individuals to under‐report sexual behaviors or minority identities, potentially resulting in misclassification. Third, because this study included only individuals presenting with AHD at first diagnosis and did not include patients diagnosed earlier in the course of infection, we are unable to compare and contrast between early and late presenters. Finally, the retrospective design precludes determination of whether patients previously accessed healthcare or were offered HIV testing prior to presentation, limiting conclusions about missed opportunities for diagnosis. Despite these limitations, this study provides a detailed characterization of patients hospitalized with AHD at first diagnosis in a high‐burden setting and highlights the demographic breadth of individuals affected.

## 5. Conclusion

Patients hospitalized with AHD at first diagnosis in Porto Alegre made up a diverse group, mostly composed of older heterosexual men and women. Fewer than one‐quarter had documented psychosocial factors typically seen as predictors of HIV risk. Notably, older age, female sex, SGM, and married/partnered status had a lower prevalence of traditional risk factors. Opt‐out HIV testing across healthcare settings in high‐mortality regions, including among individuals who may not perceive themselves to be at risk or have documented risk factors, has the potential to prevent progression to profound immunosuppression. Future studies are needed to better understand barriers to timely diagnosis and to inform more comprehensive outreach strategies.

## Author Contributions

Christopher Justin Hernandez: conceptualization, methodology, formal analysis, data curation, visualization, writing–original draft, writing–review and editing.

Pedro Moreno Fonseca: investigation, data curation, resources, writing–review and editing.

Andressa Noal: investigation, data curation, writing–review and editing.

Maxwell Zywica: investigation, data curation, writing–review and editing.

Mary Catherine Cambou: methodology, supervision, writing–review and editing.

Ivana Rosângela dos Santos Varella: investigation, resources, supervision, writing–review and editing.

Breno Riegel Santos: investigation, resources, project administration, writing–review and editing.

Marineide Gonçalves de Melo: investigation, data curation, resources, writing–review and editing.

Karin Nielsen‐Saines: conceptualization, methodology, supervision, writing–review and editing.

## Funding

This research was funded by the National Institute of Mental Health under Award Number R25MH087222.

## Disclosure

All authors reviewed and approved the final manuscript and agree to be accountable for all aspects of the work.

## Conflicts of Interest

The authors declare no conflicts of interest.

## Supporting Information

Additional supporting information can be found online in the Supporting Information section.

## Supporting information


**Supporting Information** Supporting Figure S1. Boxplots of admission CD4 cell counts (log scale) among patients newly diagnosed with advanced HIV disease during hospitalization, stratified by year (2015–2024).

## Data Availability

The data that support the findings of this study are available from the corresponding author upon reasonable request.
