# Supplementary material for: Advanced HIV Disease at First Diagnosis in South Brazil Extended Beyond Traditionally Targeted Populations: A 10‐Year Hospital‐Based Study (2015–2024)
Source: AIDS Res Treat. 2026 Jul 15;2026:6671976. doi: 10.1155/arat/6671976 (PMC13373440; doi:10.1155/arat/6671976)

Supplementary figure 1. CD4 Counts (Log-scale) at Admission Among Patients Diagnosed with Advanced HIV Disease During Hospitalization, 2015–2024


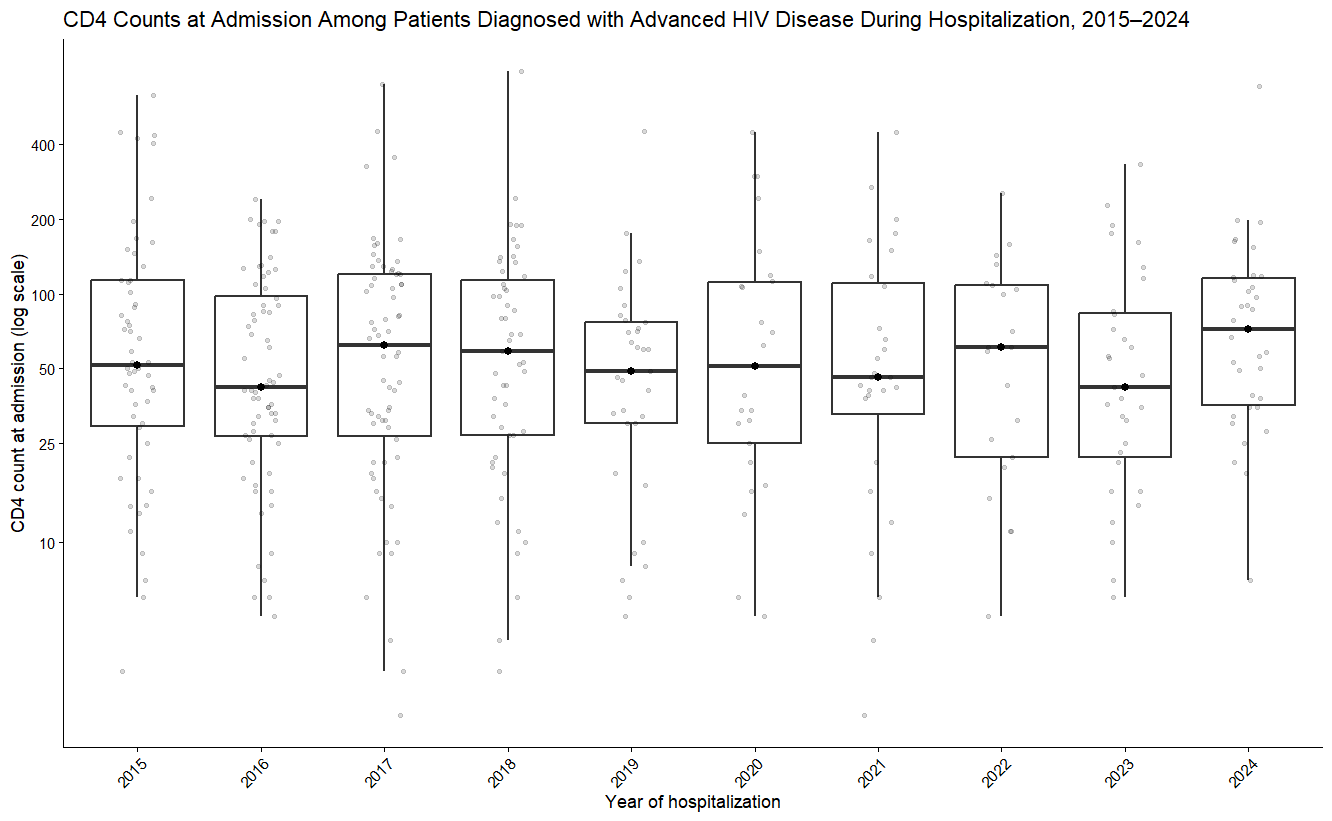

Supplement: Supplementary file 1 — Supporting Information Supporting Figure S1. Boxplots of admission CD4 cell counts (log scale) among patients newly diagnosed with advanced HIV disease during hospitalization, stratified by year (2015–2024). [file ARAT-2026-6671976-s001.docx]
